# Clinical and Therapeutic Aspects of Candidemia: A Five Year Single Centre Study

**DOI:** 10.1371/journal.pone.0127534

**Published:** 2015-05-26

**Authors:** Matteo Bassetti, Maria Merelli, Filippo Ansaldi, Daniela de Florentiis, Assunta Sartor, Claudio Scarparo, Astrid Callegari, Elda Righi

**Affiliations:** 1 Infectious Diseases Division, Santa Maria Misericordia University Hospital, Udine, Italy; 2 IRCCS San Martino IST, Department of Health Sciences, University of Genoa; Genoa, Italy; 3 USMAF, Ministry of Health, Genova, Italy; 4 Microbiology Unit, Santa Maria Misericordia University Hospital, Udine, Italy; State University of Maringá/Universidade Estadual de Maringá, BRAZIL

## Abstract

**Background:**

Candida is an important cause of bloodstream infections (BSI) in nosocomial settings causing significant mortality and morbidity. This study was performed to evaluate contemporary epidemiology, species distribution, antifungal susceptibility and outcome of candida BSI in an Italian hospital.

**Methods:**

All consecutive patients who developed candidemia at Santa Maria della Misericordia University Hospital (Italy) between January 2009 and June 2014 were enrolled in the study.

**Results:**

A total of 204 episodes of candidemia were identified during the study period with an incidence of 0.79 episodes/1000 admissions. *C*. *albicans* was isolated in 60.3% of cases, followed by *C*. *parapsilosis* (16.7%), *C*. *glabrata* (11.8%) and *C*. *tropicalis* (6.4%). Of all Candida BSI, 124 (60.8 %) occurred in patients admitted to IMW, 31/204 (15.2 %) in ICUs, 33/204 (16.2%) in surgical units and 16/204 (7.8%) in Hematology/Oncology wards. Overall, 47% of patients died within 30 days from the onset of candidemia. *C*. *parapsilosis* and *C*. *glabrata* candidemia were associated with the lowest mortality rate (36%), while patients with *C*. *tropicalis* BSI had the highest mortality rate (58.3%). Lower mortality rates were detected in patients receiving therapy within 48 hours from the time of execution of the blood cultures (57,1% vs 38,9%, *P* <0.05). At multivariate analysis, steroids treatment (OR= 0.27, p=0.005) and CVC removal (OR=3.77, p=0.014) were independently associated with lower and higher survival probability, respectively. Candidemia in patients with peripherally inserted central catheters (PICC) showed to be associated with higher mortality in comparison with central venous catheters (CVC, Short catheters and Portacath) and no CVC use. For each point increase of APACHE III score, survival probability decreased of 2%. Caspofungin (OR=3.45, p=0.015) and Amphothericin B lipid formulation (OR=15.26, p=0.033) were independently associated with higher survival probability compared with no treatment.

## Introduction

*Candida spp*. is currently the main protagonist of fungal infections in hospitalized patients, representing the fourth and sixth leading cause of nosocomial sepsis in European and US studies, respectively [1,2]. A recent American study reported Candida as the first cause of bloodstream infections (BSI) in 180 medical centres [3]. Data are available from large series of laboratory-based [4] and population-based [5] surveillance studies, as well as studies focusing on specific patient populations such as neonates [6,7], cancer [8,9], surgical [10,11] and critically ill patients [12,13]. According to the data reported by the European Centre for Disease prevention and Control (ECDC) in 2013, *Candida spp*. is actually the fifth microorganism responsible of causing sepsis in patients admitted to the ICU. Recently, an increase in incidence has been related to the complex medical and surgical procedures undertaken to prolong the survival of critically ill patients and the change in the demographic characteristics of hospitalized populations [14,15].

The epidemiology of candidemia varies according to geographical regions [16,17]. For this reason, continuous surveillance studies to monitoring incidence, species distribution, and antifungal susceptibility profiles are mandatory.

Candidemia remains associated with high crude and attributable mortality rates along withincreased cost of care and duration of hospitalization [18]. Attributable mortality has been reported to range from 5% to 71% [13,19], and crude mortality rates can be as high as 76% [20]. The increase of the attributable mortality of candidemia has driven research into the role of early diagnosis and prompt treatment initiation in order to improve outcomes [21,22]. Inadequate initial antifungal treatment has been associated with increased mortality in patients with candidemia [23]. Furthermore, intrinsic and emerging resistance to azoles represents a major challenge for empirical therapeutic and prophylactic strategies [24,25].

This study was performed to evaluate contemporary epidemiology, species distribution, antifungal susceptibly and outcome of candida BSI in an Italian hospital.

## Methods

This study was approved by the local institutional review board (Comitato Etico Unico Azienda Ospedaliera Universitaria Santa Maria della Misericordia) and written patient consent was not required because of the observational nature of this study. Patient characteristics were collected from an electronic database where records and information were anonymized and de-identified prior to analysis.

This is a retrospective, single-centre, observational cohort study conducted at Santa Maria della Misericordia hospital (Udine, Italy), a tertiary University Hospital with 960 beds and 44,000 admissions per year. All patients who developed candidemia, defined as patients with at least a positive blood culture for *Candida spp*. and a compatible clinical illness during the period January 2009—June 2014 were included in the study. Only the first episode of candidemia was recorded for each patient. Patients with “mixed candidemia”, identified as the isolation of two different *Candida* species from blood cultures performed on the same day or at a distance of at least 7 days, were also included. Clinical data were collected from the microbiological laboratory database and included underlying diseases, risk factors for candida infection (neutropenia, intravascular-devices, the administration of total parental nutrition, prior antimicrobial administration, prior antifungal therapy, recent abdominal/extra-abdominal surgery, major (surgery involving a risk to the life of the patient) and minor (an operation on the superficial structures of the body or a manipulative procedure that does not involve a serious risk) surgery, chemotherapy, radiation treatment, recent ICU admission, immunosuppressive treatment, bone marrow or solid-organ transplant), *Candida species* and susceptibility to antifungal agents, timing of antifungal administration (determined as the interval between the time when the first Candida-positive blood sample for culture was drawn and the time when the antifungal treatment was first administered to the patient) and outcome. Initial treatment was considered adequate when the infecting organism was ultimately shown to be susceptible and the dosage of antifungal used was adequate within the first 24 hours from culture positivity. The following antifungal dosages were considered adequate: fluconazole 800 mg loading dose followed by a daily dosage of at least 400 mg, liposomal amphotericin B (L-AmB) 3 mg/kg/day, amphotericin lipid complex (ABLC) 5mg/kg/day, caspofungin 70 mg loading dose followed by 50 mg/day, micafungin 100 mg/day, anidulafungin 200 mg loading dose followed by 100 mg/day.

Crude mortality rate was calculated at 30 days from blood cultures performing. No changes in the microbiological laboratory techniques at our hospital were undertaken during the study period. *Candida spp*. was isolated from blood samples using the Bactec 860 system (Becton, Dickinson, Inc., Sparks, MD). The species were identified using the API ID 32C system (bioMérieux, Marcy l'Etoile, France) or the Vitek 2 system (bioMérieux). In the cases of inconclusive results obtained by both systems, isolates were definitively identified using supplemental tests, e.g., by the presence or absence of well-formed pseudohyphae on cornmeal-Tween 80 agar and growth at 42 to 45°C. *C*. *parapislosis* strains were identified only at complex level.

Susceptibility to amphotericin B, caspofungin, fluconazole, itraconazole, and voriconazole was detected using the Sensititre YeastOne colorimetric plate (Trek Diagnostic Systems, Cleveland, OH). Until October 2011 MIC values were interpreted according to species-specific clinical breakpoints as established by the Clinical and Laboratory Standards Institute (CLSI) for amphotericin B, caspofungin, fluconazole, itraconazole (only for *C*. *albicans*), and voriconazole [26]. From November 2011 MIC values were interpreted according to species-specific clinical breakpoints as established by European Committee on Antimicrobial Susceptibility Testing (EUCAST) [27,28], updated to the last version available.

Continuous and categorical data were reported as median, 25° and 75° percentile and frequency distributions, respectively. The Wilcoxon test and χ2 test were used to determine if differences existed between groups for continuous and categorical variables, respectively. Multiple logistic regression analysis was performed to identify risk factors that were associated with hospital mortality at 3 months (JMP, SAS, NC, USA). Covariates that were significant at 0.10 in the univariate analysis were further evaluated for inclusion in multivariable regression models, using a stepwise algorithm. All tests were 2-tailed, and a p-value <0.05 was determined to represent statistical significance.

## Results

A total of 204 episodes of candidemia, corresponding to an incidence of 0.79 cases / 1000 admissions, was recorded during the study period. The incidence of candidemia corresponded to 0.70–0.84–0.77–0.93–0.72 episodes /1000 admissions for 2009, 2010, 2011, 2012, and 2013, respectively. The demographic and clinical characteristics of the patients are summarized in Table 1. The most frequently represented age group was 61 to 80 years of age (46.9% of cases): more than 73% of candidemia were recorded in patients older than 60 years. During the study period, the mean age of the population rose from 61.2 years in 2009 to 73.5 years in 2014. Over 87% of the patients (178/204) had one or more comorbidities at the time of the diagnosis of candidemia. Specifically, 34.8% presented a solid organ malignancy, 27.9% were diabetics, 12.3% presented liver diseases, 8.3% renal failure, and 4.9% a haematological disease. Among immunosuppressive conditions, 55 (26.9%) received steroid therapy, 28 (13.2%) underwent chemo and / or radiotherapy, 7 (2.9%) received monoclonal antibodies and 9 (3.9%) received other immunosuppressive agents. Seventy patients (34.3%) underwent surgery in the two months prior to candidemia; of these, 31 had undergone major surgery, 18 a minor surgical procedures, and 21 had extra—abdominal operations. Nineteen/70 (24.3%) had a re-intervention in the two months preceding the diagnosis of candidemia. Forty-nine (24%), were hospitalized in the ICU in the month prior to the diagnosis of candidemia. A total of 140 (69.1%) patients received parental nutrition. An intravascular device was present in 84.4% of the patients on the candidemia was diagnosed. Ninety-one percent of the patients with candidemia had received at least one antibiotic for ≥ 7 days during the month before candidemia diagnosis. An episode of concomitant bacteraemia was detected in 18.8% of patients; of these, 72% was caused by Gram-positive bacteria, 25% by Gram-negative bacteria and by mixed bacterial flora in the remaining cases.

**Table 1:** Patients’ features and ward distribution among candida species (n = 204).

	*Candida* species					
	*C*. *albicans (n = 123)*	*C*. *parapsilosis (n = 34)*	*C*. *glabrata (n = 24)*	*C*. *tropicalis (n = 13)*	*Others (n = 10)*	All (n = 204)
	60.2%	16.7%	11.8%	6.4%	5.2%	100%
**Patients characteristics**						
Male sex, n (%)	74 (60)	20 (59)	15 (63)	7 (54)	7 (70)	**118 (58)**
Mean age (SD)	67 (±21)	71 (±18)	71 (±22)	68 (±16)	53 (±18)	**67(±20)**
**Risk factors**						
Recent ICU admission	31 (25.2)	6 (17.6)	8 (33.3)	0	4 (40)	49 (24)
Central venous catheter (CVC)	105 (85.4)	30 (88.2)	17 (70.8)	11 (84.6)	9 (90)	172 (84.4)
Parenteral nutrition	87 (70.7)	27(79.4)	13 (54.2)	8 (66.7)	6 (60)	141 (69.1)
Antibiotic treatment in the previous 30 days	117 (95.1)	25 (73.5)	23 (95.8)	12 (92.3)	9 (90)	186 (91.2)
Major abdominal surgery	24 (19.5)	5 (14.7)	2 (8.3)	1 (7.7)	1(10)	31 (16.2)
Minor abdominal surgery	11 (8.9)	1 (2.9)	4 (16.7)	2 (11)	0	18 (8.8)
Extra-abdominal surgery	15 (12.2)	3 (8.8)	1(4.2)	2 (20)	0	21 (10.3)
Solid tumor	40 (32.5)	11 (32.3)	10 (41.7)	6 (8)	4 (40)	71 (34.8)
Haematological malignancies	5 (4,1)	1 (2,9)	0	3 (30)	1 (10)	10 (4.9)
Chemo/radio-therapy	13 (10.6)	5 (14.7)	1 (4.2)	5(19)	3 (30)	27 (13.2)
Diabetes mellitus	34 (27.6)	8 (23.5)	8 (33.3)	3 (5)	4 (40)	57 (27.9)
Haemodialysis	14 (11.4)	2 (5.9)	1(4.2)	0	1 (10)	18 (8.8)
Steroid treatment	30 (24,4)	10 (29.4)	8 (33.3)	4 (7)	3 (30)	55 (26.9)
**Hospital Units**						
Medical Units	70 (56.9)	23 (67.6)	15 (62.5)	7 (53.8)	6 (60)	121 (59.3)
Surgical Units	25 (20.3)	2 (5.9)	4 (16.7)	1 (7.7)	1 (10)	33 (16.2)
Hematology/Oncology	5 (4)	4 (11.8)	1 (4.2)	5 (38.5)	2 (20)	17 (8.3)
ICU	23 (18.7)	5 (14.7)	4 (16.7)	0	1 (10)	33 (16.2)

**Fig 1:**
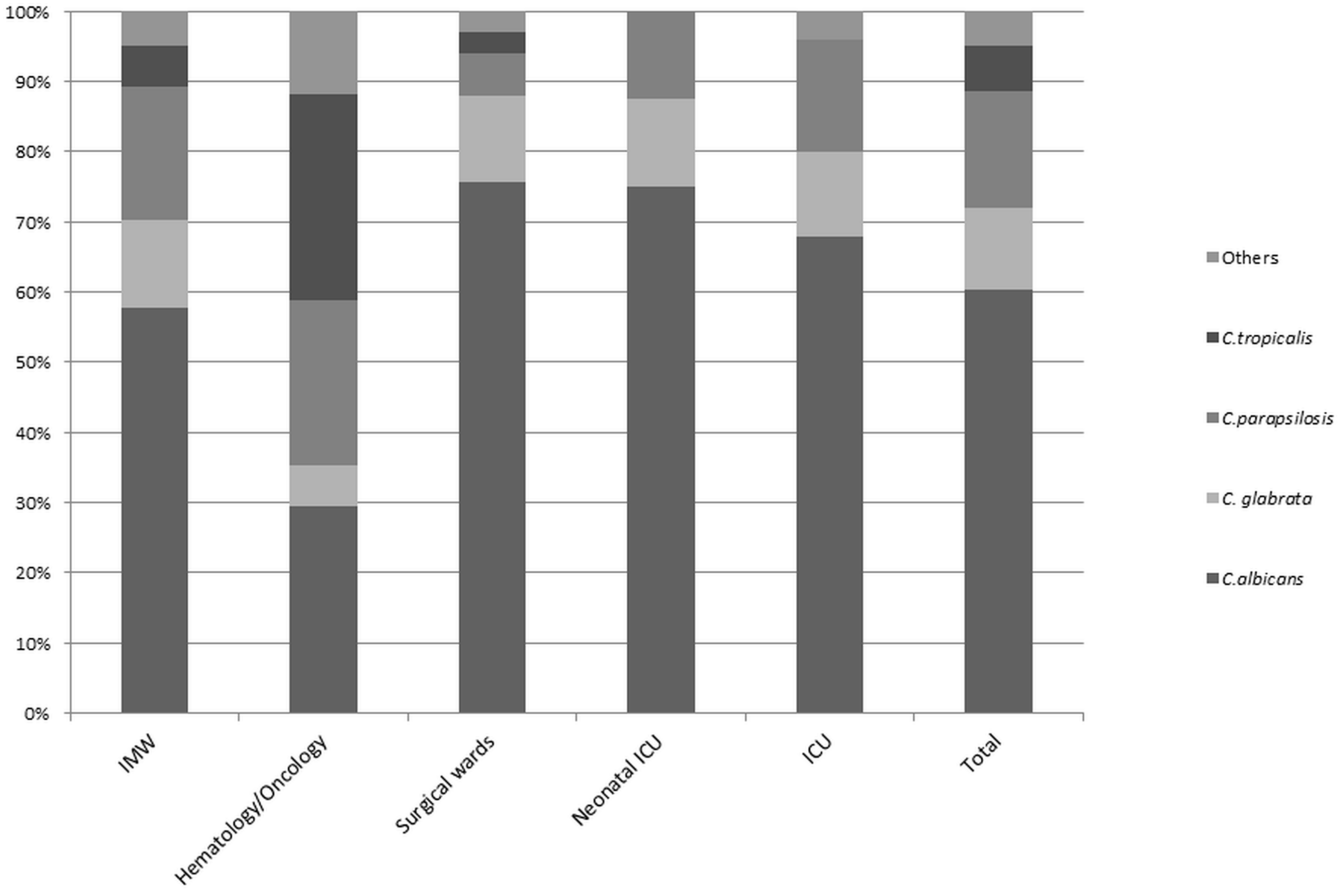
Candida spp. distribution expressed as percentage of total candida species in various hospital wards.

One hundred and twenty-four cases (60.8%) occurred in patients admitted to IMW, 31/204 (15.2%) in ICUs, 33/204(16.2%) in surgical units, and 16/204 (7.8%) in Hematology/Oncology wards. Candidemia distribution by hospital ward is shown in Fig 1.

Overall, *C*. *albicans* (CA) was the most common pathogen, accounting for 60% of total cases, followed by *C*. *parapsilosis* (17%), *C*. *glabrata* (12%), *C*. *tropicalis* (6%) and others (5%). The proportion of CA versus non-albicans Candida (CAN) changed over the years: CA was responsible for 66.7% of candidemia in 2009, 67.7% in 2010, 61.7% in 2011, 43.2% in 2012, 61.7% in 2013, and 68.4% in 2014.

Table 2 shows the results of the *in vitro* activity of 3 systemically active antifungal agents tested against BSI isolates of *Candida spp*. based on CLSI and EUCAST breakpoints. The rate of susceptibility to fluconazole was 98.4% and 99.2% for *C*. *albicans* according to CLSI and EUCAST breakpoints respectively, and 88.2% for *C*. *parapsilosis* according to CLSI breakpoints. Decreased susceptibility to fluconazole was mostly seen with *C*. *glabrata* (83.3%).

**Table 2:** MIC_50_, MIC_90_, and susceptibility of Candida strains to antifungals.

*Candida species* N° (% isolates)	Antifungal agent	MIC range (μg/mL)	MIC 50 (μg/ml)	MIC 90 (μg/ml)	N° (%) of resistant or SDD isolates	
					CLSI	EUCAST
***C*. *albicans* 123 (60.3%)**	Fluconazole	0.12–256	0,25	1	2 (1.6)	1 (0.8)
	Caspofungin	0.008–1	0,06	0,06	0	N/A
	Amphotericin B	0.25–1	0,125	0,5	N/A	0
***C*. *parapsilosis* 34 (16.7%)**	Fluconazole	0.25–256	1	4	1 (2.9)	3 (8.8)
	Caspofungin	0.03–2	0.5	0.5	2 (5.9)	NR
	Amphotericin B	0.016–0.5	0.06	0.25	N/A	0
***C*. *glabrata* 24 (11.8%)**	Fluconazole	1–32	8	16	0	4 (16.7)
	Caspofungin Amphotericin B	0.06–0.5 0.06–1	0.12 0.125	0.125 0.5	0 N/A	N/A 0
	Amphotericin B	0.06–1	0.125	0.5	N/A	0
***C*.*tropicalis* 13 (6.4%)**	Fluconazole	0.5–4	1	4	1(7.7)	1(7.7)
	Caspofungin	0.03–0.25	0.06	0,125	0	Na
	Amphotericin B	0.12–0.5	0.25	0.5	N/A	0
***Others* 10 (5%)**	Fluconazole	0.12–32	1	32	2 (20)	1(10)
	Caspofungin	0.12–05	0.25	0.25	0	N/A
	Amphotericin B	0,015–0,25	0,06	0,25	N/A	0
**All *Candida spp*. 204**	Fluconazole	0.12–256	0.5	8	7 (3.4)	10 (4.9)
	Caspofungin	0.008–2	0.06	0.5	0	N/A
	Amphotericin B	0.015–1	0.125	0.5	0	0

N/A: breakpoint not available, NR: not recommended

Type of treatment and outcome data were available for 190 patients. A total of 108 (56.8%) patients received adequate antifungal treatment within 48 hours after collection of blood cultures. Ninety patients (47.4%) died within 30 days following the diagnosis of candidemia.

When 30-day mortality was compared among different wards, significantly higher rates were observed in IMW compared to surgical wards (*P* = 0.002) and to all the other wards (*P* = 0.009). Thirty-day mortality rates for the population that received antifungal therapy within 48 hours from collection of blood cultures was 38.9% compared to 57.1% observed in the population in which the timing was > 48 hours (*P* = 0.03).

Empirical antifungal treatment was different in patients younger and older than 65 years (p = 0,001) (Table 3). A higher proportion of younger patients received caspofungin treatment compared to older patients [21/71 (29.6%) vs. 23/103 (17.3%) respectively, P = 0.001].

**Table 3:** Comparison of clinical characteristics between patients under and over 65 years old.

	Under 65 N° (%) (n. = 71)	Over 65 N° (%) (n. = 133)	p-value
**Gender male**	45 (63.4)	78 (58.7)	0.433
**Median age (range)**	53 (37–60)	78 (71–84.5)	**<0.001**
**Hospital wards**			
**Internal medicine Wars**	39 (54.9)	85 (63.9)	NS
**Surgical Wards**	14 (19.7)	19 (14.3)	NS
**Intensive Care Units**	13 (18.3) 5 (7)	18 (13.5) 11 (8.3)	NS
**Onco/hematological Units**	5 (7)	11 (8.3)	NS
**CVC**			
**No CVC**	5 (7)	27 (20.3)	**<0.001**
**Short catheter**	36 (50.7)	49 (36.8)	**<0.001**
**Umbilical arterial catheter**	5 (7)	0	NS
**Midline**	3 (4.2)	13 (9.8)	NS
**Peripherally Inserted Central Catheter (PICC)**	9 (12.7)	27 (20.3)	**<0.001**
**Portacath**	13 (18.3)	17 (12.8)	**<0.001**
**Total Parenteral Nutrition**	51 (71.8)	89 (66.9)	0.471
**Percutaneous Enteral Nutrition**	4 (5.6)	12 (9)	0.735
**Antibiotic treatment**	64 (90.1)	121 (91)	0.845
**Abdominal surgery**	9 (12.7)	23 (17.3)	0.57
**Extra-abdominal surgery**	10 (14.1)	11 (8.3)	0.193
**Reoperation**	10 (14.1)	9 (6.8)	0.087
**Solid cancer**	28 (39.4)	43 (32.3)	0.31
**Hematological cancer**	6 (8.5)	4 (3)	0.086
Chemo/radiotherapy*	20 (28.2)	8 (6)	**<0.001**
**Monoclonal antibodies**	5 (7)	2 (1.5)	**0.038**
**Diabetes**	24 (33.8)	33 (24.8)	0.172
**Liver disease**	14 (19.7)	13 (9.8)	**0.046**
**Hemodialysis**	8 (11.3)	10 (7.5)	0.368
**Steroids**	24 (33.8)	31 (23.3)	0.108
**Other immunosuppressant therapy**	6 (8.5)	3 (2.39	**0.04**
**Mechanical ventilation**	20 (28.2)	11 (8.3)	**<0.001**
**Apache III**	36 (24.5–56.5)	39 (35–52.75)	0.12
**Vasopressor use**	15 (21.1)	12 (9)	**0.015**
**HIV**	4 (5.6)	0	**0.006**
**CVC removal within 24h**	11 (15.5)	21 (15.8)	0.956
**Empiric antifungal treatment**	66 (93)	102 (76.7)	**0.001**
**Empirical treatment**			
**Fluconazole**	37 (52.1)	73(54.9)	**NS**
**Voriconazole**	2(2.8)	0	**NS**
**Amphotericin B lipid-formulations**	2 (2.8)	1 (1.5)	**NS**
**Caspofungin**	21 (29.6)	23 (17.3)	**0.001**
**Other echinocandins**	3 (2)	4 (3)	**NS**
**Empiric antifungal treatment**	66 (93)	102 (76.7)	**0.001**
**Definitive treatment**			
**Fluconazole**	9(12.7)	10 (7.5)	**NS**
**Voriconazole**	2(2.8)	2 (1.5)	**NS**
**Amphotericin B lipid-formulations**	5 (7)	1 (0.8)	**NS**
**Caspofungin**	11 (15.5)	18 (13.5)	**NS**
**Other echinocandins**	2 (2.8)	2 (1.5)	**NS**
**Timing of therapy**			
**Within 24h**	20 (28.2)	37 (27.8)	0.96
**Between 24 and 48h**	9 (12.7)	16 (12)	0.893
**More than 48 H**	2 (2.8)	7 (5.3)	0.418
**30-day mortality**	30 (42.2)	74 (55.6)	0.071

CVC: central venous catheter

Table 4 summarizes the significant differences between patients with candidemia who died within 3 months from the diagnosis of candidemia compared with survivors at univariate analysis. Patients who died were significantly older than survivors (73 vs. 67.5 years, P = 0.005). Compared to patients who died, the survivors had significant lower APACHE III scores (median 37 vs. 41, p = 0.003). At univariate analysis, abdominal surgery (p<0,001), re-operation (p = 0,014) and CVC removal (p<0,001) were more frequent in survivors, while steroids treatment was more often present in patients who died (p = 0.006). Distribution of CVC was significantly different in patients with different mortality outcomes (p = 0.002): in particular, peripherally inserted central catheters (PICC) use was more frequently used in patients who died compared to survivors (25,8% vs. 4%

**Table 4:** Characteristics of survivors (N = 76) compared with patients with candidemia who died (N = 128).

	Alive N° (%) (n. = 76)	Deaths N° (%) (n. = 128)	p-value
Gender (male)	32 (42.1)	49 (38.3)	0.589
Avarage age (range)	67.5 (53–78)	73 (61.25–83)	**0.005**
Over 65 years	44 (57.9)	89 (69.5)	0.091
Hospital wards			
Internal medicine Wars	39 (51.3)	85 (66.4)	NS
Surgical Wards	17 (22.4)	16 (12.5)	NS
Intensive Care Units	13 (17.1)	18 (14.1)	NS
Onco/hematological Units	7 (9.2)	9 (7)	NS
CVC			
No CVC	13 (17.1)	19 (14.8)	**NS**
Short catheter	42 (55.3)	43 (33.6)	**0.002**
Umbilical arterial catheteR	2 (2.6)	3 (2.3)	**NS**
Midline	4 (5.3)	12 (9.4)	**NS**
Peripherally Inserted Central Catheter	3 (4)	33 (25.8)	**0.002**
Portacath	12 (15.8)	18 (14.1)	**NS**
Total Parenteral Nutrition	49 (64.5)	91 (71.1)	0.324
Percutaneous Enteral Nutrition	7 (9.2)	9 (7)	0.575
Antibiotic treatment	69 (90.8)	116 (90.6)	0.969
Abdominal surgery	21 (27.6)	11 (8.6)	**<0.001**
Extra-abdominal surgery	6 (7.9)	15 (11.7)	0.385
Re-operation	12 (15.8)	7 (5.5)	**0.014**
Solid cancer	27 (35.5)	44 (34.4)	0.867
Hematological cancer	6 (7.9)	4 (3.1)	0.127
Chemo/radiotherapy*	8 (10.5)	20 (15.6)	0.21
Monoclonal antibodies	2 (2.6)	5 (3.9)	0.629
Diabetes	16 (21.1)	41 (32)	0.091
Liver disease	9 (11.8)	18 (14.1)	0.651
Hemodialysis	10 (13.2)	8 (6.3)	0.093
Steroids	12 (15.8)	43 (33.6)	**0.006**
Other immunosuppressant therapy	4 (5.3)	5 (3.9)	0.648
Mechanical ventilation	11 (14.5)	20 (15.6)	0.824
Apache III	37 (25.25–44.75)	41 (33–56)	**0.003**
Vasopressor use	7 (9.2)	20 (15.6)	0.191
HIV	2 (2.6)	2 (1.6)	0.59
Bacteremia	14 (18.4)	24 (18.8)	0.953
CVC removal in 24h	21 (27.6)	11 (8.6)	**<0.001**
Empirical treatment			
No treatment	7 (9.2)	9 (22.7)	0.075
Fluconazole	44 (57.9)	29 (22.7)	NS
Voriconazole	1 (1.3)	1 (0.8)	NS
Caspofungin	18 (23.7)	26 (20.3)	NS
Other echinocandins	3 (4)	4 (3.1)	NS
Amphotericin B lipid formulation	3 (4)	1 (0.8)	NS
Definitive treatment			
No treatment	42 (55.3)	100 (78.1)	**0.029**
Fluconazole	10 (13.2)	9 (7)	**NS**
Caspofungin	16 (21.1)	13 (10.2)	**0.029**
Amphotericin B lipid formulation	3 (4)	3 (2.3)	**NS**
Timing of therapy			
Within 24h	24 (31.6)	33 (25.8)	0.372
Between 24 and 48h	11 (14.5)	14 (10.9)	0.456
More than 48 h	4 (5.3)	5 (3.9)	0.648

The final logistic regression model included CVC type, steroids treatment, Apache III score, CVC removal and definitive treatment. Steroids treatment (OR = 0.27, p = 0.005) and CVC removal (OR = 3.77, p = 0.014) were independently associated with lower and higher survival probability, respectively (Table 5). PICC showed to be associated with higher mortality in comparison with more used CVCs (Short catheter, Portacath) and no CVC use. Fo each point increase of APACHE III score there was a 2% decrease of survival probability. Caspofungin (OR = 3.45, p = 0.015) and Amphothericin B lipid formulation (OR = 15.26, p = 0.033) were independently associated with significantly higher survival probability in comparison with no treatment.

**Table 5:** Multivariate analysis of risk factors for 30-day mortality.

	Effect likelihood ratio test			Survival Probability	
	L-R chi square	p-value	Significant ratio	OR(95% C.I.)	
**CVC**	11.63	0.02	Short catheter vs. PICC	7 (1.8–38.17)	**0.004**
			Portacath vs. PICC	8.19 (1.86–48.27)	**0.005**
			no CVC vs. PICC	8.41 (1.85–50.86)	**0.005**
**Steroids**	7.68	0.005	Steroids vs. no steroids treatment	0.27 (0.09–0.69)	
**Apache III**	4.1	0.043	Per unit increase	0.98 (0.95–0.99)	
**CVC removal**	6.04	0.014	Removed vs. not removed	3.77 (1.3–11.76)	
**Definitive treatment**	15.81	0.007	caspofungin vs no treatment	3.49 (1.28–10)	**0.015**
			amphotericin B lipid formulation vs. no treatment	15.26 (1.25–366.13)	**0.033**

## Discussion

An increased incidence of candidemia in nosocomial settings has been shown by many studies in the last fifteen years [1,29]. Our study shows a substantial stability in the incidence over the past 5 years. Candidemia was more frequently diagnosed among patients aged 61 to 80 years, with a progressive increase of the mean age over time. We also observed a progressive, gradual rise of the population mean age, with an overall increase of more than 10 years during the 5 years of observation. The increase in mean age partially differs from other studies where a mean age 60 years was reported with no significant changes in the last decade [12,30]. However, our age population data aligns perfectly with other Italian studies [16,19,31,32]. As previously reported, our data suggests that the increase in the incidence of candidemia is related not only to an increased number of immunocompromised patients, but also to the aging of the population [31].

*C*. *glabrata* and *C*. *parapsilosis* BSI were more likely to occur in older patients. Conversely, candidemia caused by less common species, such as *C*. *krusei* and *C*. *famata*, were more often associated with younger age. We reported similar or higher proportions of immunocompromised patients compared with other studies [4,12,25]. Furthermore, high proportions of patients received broad spectrum antibiotics and steroid therapy prior to hospitalization with candidemia. As previously reported, we also observed high percentages of patients with parenteral nutrition and central vascular access, especially in *C*. *parapsilosis* fungemia [33]. As confirmed by our study, the incidence of candidemia in IMW is increasing [31,34] due to the increase in the mean age of hospitalized patients, a more extensive use of antibiotics, steroid therapy, and invasive procedures performed outside the ICUs [34].

Although there is an increasing evidence of the progressive rise of NCA, *C*. *albicans* still represents the most isolated species in Europe [19,35–37]. Although *C*. *albicans* was the main isolate in our hospital, the distribution varied considerably according to the ward. Similarly to other studies, *C*. *albicans* was the prevalent species in surgical wards, but not in haematology/oncology where CNA were prevalent [32]. The high proportion of *C*.*albicans BSI* observed in surgical units diverges from what shown by previous studies [2,32]. This could be related to a limited use of fluconazole prophylaxis performed in these units as previously reported [36]. Among CNA, *C*. *parapsilosis* was mostly isolated in IMW where an extensive use of intravascular-devices is widely practiced and *C*. *krusei* was more common among patients with hematologic disorders [38] who frequently receive azole-based prophylaxis or treatment.

Non-susceptibility to fluconazole (8.3%) was slightly higher than the one reported by European (6.3%) and North American (6.6%) studies, but appeared lower compared to other Italian studies where resistances up to 25% have been documented [16,34].

The presence of a concomitant bacteraemia in around 20% of cases was lower than the one reported in another study [39].

We showed 30-day mortality rates of 47.4%, similar to the ones reported by a recent study conducted in 5 Italian and Spanish academic hospitals [18]. During the study period, we did not observe substantial changes in both source control (e.g., intravascular device removal) and timing for antifungal treatment initiation, thus suggesting that the management of candidemia at our institution can still be improved. Antifungal therapy timing is crucial and is known to significantly impact the mortality of patients with candidemia [18,21,22]. We observed a significant difference in mortality between the patients who received antifungal therapy within 48 hours of collection of a blood culture and those who were treated after 48 hours. In particular, medical wards had the lowest rates of timely initiation of antifungal therapy and presented a significantly higher mortality if compared to surgical wards (*P* = 0.002).

Our study supports the importance of the source control (e.g., catheter removal) in patients with candidemia. Source control has previously been shown to be an important determinant of outcome for patients with serious infections attributed to Candida species [40]. Although controversy exists regarding the need to remove all central venous catheters in candidemic patients, the recent European guidelines support the early catheter removal [41].

Our results align with a recent patient-level review of candidemia trial that reported improved clinical outcomes in patients receiving an echinocandin [42].

In conclusion, this study confirms that candidemia is an important cause of morbidity and mortality, especially among elderly patients admitted to IMW. Early identification of risk factors associated with this disease is necessary to reduce its incidence, and a timely management is crucial to improve the outcome.
